# Methicillin-Susceptible ST398 *Staphylococcus aureus* Responsible for Bloodstream Infections: An Emerging Human-Adapted Subclone?

**DOI:** 10.1371/journal.pone.0028369

**Published:** 2011-12-05

**Authors:** Anne-Sophie Valentin-Domelier, Myriam Girard, Xavier Bertrand, Jérémie Violette, Patrice François, Pierre-Yves Donnio, Daniel Talon, Roland Quentin, Jacques Schrenzel, Nathalie van der Mee-Marquet, for the Bloodstream Infection Study Group of the Réseau des Hygiénistes du Centre (RHC)

**Affiliations:** 1 Service de Bactériologie et Hygiène, Hôpital Trousseau, Centre Hospitalier Universitaire, Tours, France; 2 Genomic Research Laboratory, University of Geneva Hospitals, Geneva, Switzerland; 3 Service d'Hygiène, Centre Hospitalier Universitaire, Université de Franche-Comté, Besançon, France; 4 UMR 6249 Chrono-environnement, Université de Franche-Comté, Besançon, France; 5 Service de Bactériologie et Hygiène Hospitalière, Centre Hospitalier Universitaire, Université de Rennes 1, Rennes, France; 6 EA1254 Microbiologie-Risques Infectieux, Université de Rennes 1, Rennes, France; 7 Clinical Microbiology Laboratory, University of Geneva Hospitals, Geneva, Switzerland; 8 Réseau des Hygiénistes du Centre, Centre Hospitalier Universitaire, Tours, France; University of Edinburgh, United States of America

## Abstract

In the course of an annual 3-month bloodstream infections (BSI) survey conducted during a four-year period in 31 healthcare institutions located in three noncontiguous French regions, we report 18 ST398 *Staphylococcus aureus* BSI. ST398 BSI incidence showed a seven-fold increase during the study period (0.002 per 1,000 patient days in 2007 vs. 0.014 in 2010). ST398 BSI isolates differed from the pig-borne multiresistant clone: 17/18 BSI isolates were methicillin susceptible and none was of t011, t034 or t108 pig-borne *spa-*types. ST398 BSI isolates had homogenous resistance patterns (15/18 with only Ery^r^) and prophagic content (all harboured the *hlb*-converting Sau3int phage). The clustering of BSI and pig-borne isolates by *spa-*typing and MLVA, the occurrence of Sau3int phage in BSI isolates and the lack of this phage in pig-borne isolates suggest that the emergence of BSI isolates could have arisen from horizontal transfer, at least of the Sau3int phage, in genetically diverse MSSA ST398 isolates. The acquisition of the phage likely plays a role in the increasing ability of the lysogenic ST398 isolates to colonize human. The mode of acquisition of the non pig-borne ST398 isolates by our 18 patients remains unclear. ST398 BSI were diagnosed in patients lacking livestock exposure and were significantly associated with digestive portals of entry (3/18 [16.7%] for ST398 vs. 19/767 [2.5%] for non ST398 BSI; p = .012). This raises the question of possible foodborne human infections. We suggest the need for active surveillance to study and control the spread of this human-adapted subclone increasingly isolated in the hospital setting.

## Introduction

Primarily described in Europe, ST398 methicillin-resistant *S. aureus* (MRSA) is now a worldwide threat associated with livestock, their human contact and food products [1]. Association with animals or animal farming is recognized as major risk factor for infections associated with ST398 MRSA. Skin, soft tissue and invasive infections are mainly associated with isolates of *spa-*types t011, t034 or t108 [1]. Beside this, there are few recent reports of infections caused by ST398 *S. aureus* in persons lacking livestock-associated (LA) risk factors. Most of these involve MSSA of *spa-*type t571 [2]-[4]. Previous characterization of ST398 pig-borne isolates have indicated that most are MRSA strains of *agr* type I, do not contain any genes encoding the major staphylococcal virulence factors, harbour genes involved in the colonization and the early stages of infections such as the *cna* gene and the hyaluronidase gene and appear deficient in the type I restriction system (*hsdS-hsdR*) [5]. This is a system whose main function is to limit horizontal gene transfer. Unlike the pig-borne strains, ST398 isolates associated with infections in persons lacking LA risk factors are mostly MSSA strains, harbor *cadC-cadM* prophagic genes shared with USA300 (ST8) virulent clone, and include remnants of SCC*mec* cassette [2], [4].

Reporting of *Staphylococcus aureus* bloodstream infections (BSI) is often mandatory. Reduction of hospital-acquired (HA) MRSA BSI rates is a performance target [6]. A longitudinal survey of BSI under way since 2002 in the region Centre of France showed a decreasing incidence rate of *S. aureus* BSI from 2002 to 2006 and an increased incidence of methicillin-sensitive *S. aureus* (MSSA) BSI since 2006 [7]. The emergence of MSSA ST398 BSI cases was described in the same context in 2009 [2].

To assess the incidence of ST398 BSI cases at a national level, we screened for ST398 all *S. aureus* BSI cases diagnosed into three noncontiguous French regions in the course of the annual BSI survey between 2007 and 2010. We characterized the identified ST398 isolates by antimicrobial susceptibility testing and *luk*, *tst*, *eta*, and *etb* gene detection.Using pulsed-field gel electrophoresis (PFGE) typing, multiple-locus variable-number tandem-repeat analysis (MLVA) and prophagic gene content analysis, we studied the genetic diversity of the population of BSI isolates and compared BSI isolates with a set of 12 isolates representative of the major “classical” pig-borne clones.

## Materials and Methods

### BSI epidemiological survey method

#### Study population

Thirty-one healthcare institutions (HCIs) located into three noncontiguous French regions and comprising a total of 9,329 acute-care beds, participated in the study: three university hospitals, –one located in the Centre region (1309 beds), one in the Franche-Comté region (1112 beds) and one in the Bretagne region (1330 beds)–, and 28 HCIs located in the Centre region and comprising six general hospitals (364 to 580 beds), 10 private clinics (68 to 344 beds) and 12 local hospitals (58 to 141 beds). *Study design*. Following methods and a study design previously reported [7], a BSI surveillance program which involves an annual 3-month survey of all cases of BSI, and a microbiological study of *S. aureus* strains isolated from BSI cases have been conducted in 2007, 2008, 2009 and 2010. The survey covered 2,436,583 acute-care patient days (PD). *S. aureus* BSI were defined according to international definitions (CDC). The variables studied included patient age and sex, portal of entry (skin [primitive cutaneous form or superinfection of a skin breach], surgical site, lungs, urine, intravascular device, or digestive tract), BSI origin (community-acquired or nosocomial), death within 7 days of BSI diagnosis, and duration of hospital stay. The data were analyzed with Epi Info version 6 software. Ethical approval of the BSI surveillance program was obtained at the national level from the Réseau Alerte Investigation Surveillance des Infections Nosocomiales (RAISIN).

### Bacteriology

BSI-associated *S. aureus* isolates were collected during each of the four three-month periods and conserved at -80°C. The isolates were identified as *S. aureus* according to routine methods in each laboratory. All *S. aureus* isolates were then sent to the laboratory of the Réseau des Hygiénistes du Centre. Twelve isolates representative of the major european pig-borne ST398 MSSA and MRSA strains, and three unrelated virulent strains were also studied. *Antimicrobial susceptibility testing.* The disk diffusion method (Bio-Rad, France) was used to test the antibiotic susceptibilities of all isolates. According to the French National Committee recommendations [8], the antibiotics tested were penicillin G, cefoxitin, erythromycin, lincomycin, pristinamycin, tetracycline, kanamycin, tobramycin, gentamicin, rifampin (rifampicin), fusidic acid, fosfomycin, pefloxacin, cotrimoxazole, vancomycin, and teicoplanin. *ST398 PCR.* Detection of ST398 among *S. aureus* isolates was performed using a PCR technique targetting *sau1-hsdS1* gene according to a procedure previously described by Stegger *et al.* [5]. *MLST.* We confirmed the assignation of the isolates positive with ST398 PCR by using MLST according to the procedure described by Enright *et al.* [9]. The ST398 isolates were further characterized. *Virulence factors.* We sought sequences corresponding to *lukS-PV* and *lukF-PV*, encoding Panton-Valentine leukocidin, to *tst*, encoding TSST-1, and to the *eta* and *etb* genes, encoding exfoliatins A and B, respectively, by PCR amplification using a procedure previously described by Jarraud *et al.* [10]. *spa*-types were determined for all isolates as previously described and were assigned through the *spa-*type database www.ridom.de/spaserver [11]. *Phage integrase multiplex PCR.* Sequences corresponding to seven prophagic genes were sought by PCR amplification using a procedure previously described by Goerke *et al.* [12]. *VNTR analysis.* According to François *et al.* [13], multiplex PCR amplifications with eight primer pairs that target gene regions with variable numbers of tandem repeats were resolved by microcapillary electrophoresis and automatically assessed by cluster analysis to compare ST398 BSI isolates with pig-borne ST398 MSSA and MRSA isolates and reference strains. *DNA macrorestriction and PFGE.* PFGE was performed according to the previously described procedure [14]. *Apa*I, *Eag*I and *Crf9*I PFGE patterns were analyzed with the Gelcompar package (Gelcompar software, Applied Maths, France) and interpreted according to international recommendations [14].

### Statistical data

Chi-square tests and Fisher's exact test (two-tailed) were used to test associations, and a *P* value of 0.05 was considered significant.

## Results

During the study period, 858 *S. aureus* BSI were diagnosed in 566 males and 292 females. BSI were associated with MSSA in 670 cases (78.1%) and MRSA in 188 (21.9%). Among the 858 BSI, 556 were hospital-acquired (HA) (64.8%), resulting in a mean incidence of 0.352 per 1,000 PD (table 1). The HA-BSI incidence increased 7.1% during the study (0.239 in 2007 *vs* 0.256 in 2010, table 1) due mostly to the 20.4% increase of MSSA BSI incidence (0.167 in 2007 *vs* 0.201 in 2010) whilst MRSA declined by 23.5% (0.071 in 2007 *vs* 0.055 in 2010). The portal of entry was known in 667 cases (77.7%). The increase in *S. aureus* HA-BSI incidence was mainly due to MSSA HA-BSI associated with surgical sites (0.035 in 2007 *vs* 0.043 in 2010) or intravenous devices (0.050 in 2007 *vs* 0.067 in 2010) (table 1). By contrast, incidence of IVD-associated and surgery-associated MRSA BSI did not vary during the study period. Among 755 patients (88.0%) whose survival outcome was documented in the database, 80 deaths (10.6% mortality) were documented.

**Table 1:** Characteristics of MRSA and MSSA responsible for BSI during the 5-year period.

	Year	Number	BSI	Sex of the patient		Portal of entry of the BSI								Death^2^
		of BSI	incidence	M	F	Skin	Surgical Site	Pulmonary	Urinary	IVD^1^	Digestive	others	Unknown	within 7 days
MSSA		670	0.275	442	228	136	82	57	43	128	21	51	152	54 (83 nk)
CA	all	251	0.103	162	89	86		27	13		6	33	86	21 (27 nk)
	2007	50	0.083	28	22	12		6	2		3	12	15	3 (2 nk)
	2008	64	0.103	42	22	24		7	5			3	25	6 (2 nk)
	2009	66	0.105	45	21	23		4	5		3	11	20	3 (10 nk)
	2010	71	0.121	47	24	27		10	1			7	26	9 (13 nk)
HA	all	419	0.172	280	139	50	82	30	30	128	15	18	66	33 (56 nk)
	2007	101	0.167	69	32	14	21	6	10	30	3	3	14	15 (1 nk)
	2008	97	0.156	68	29	12	18	7	6	27	3	5	19	11 (1 nk)
	2009	103	0.164	73	30	12	18	9	7	32	4	4	17	4 (26 nk)
	2010	118	0.201	70	48	12	25	8	7	39	5	6	16	3 (28 nk)
MRSA		188	0.077	124	64	45	18	21	25	30	5	5	39	26 (20 nk)
CA	all	51	0.021	36	15	18		7	7			2	17	4 (7 nk)
	2007	9	0.015	5	4	1		1	2				5	2 (1 nk)
	2008	15	0.024	9	6	5		3	3				4	2
	2009	13	0.021	11	2	5			2			1	5	0 (4 nk)
	2010	14	0.024	11	3	7		3				1	3	0 (2 nk)
HA	all	137	0.056	88	49	27	18	14	18	30	5	3	22	22 (13 nk)
	2007	43	0.071	28	15	6	6	4	4	11	3	2	7	7 (1 nk)
	2008	33	0.053	20	13	10	6	4	2	5		1	5	5 (1 nk)
	2009	29	0.046	19	10	5	2	3	7	6	1		5	5 (5 nk)
	2010	32	0.055	21	11	6	4	3	5	8	1		5	5 (6 nk)
All		858	0.352	566	292	181	100	78	68	158	26	56	191	80 (103 nk)
CA		302	0.124	198	104	104		34	20		6	35	103	25 (34 nk)
HA		556	0.228	368	188	77	100	44	48	158	20	21	88	55 (69 nk)

1 Intra-Venous Devices

2 nk not known.

Seven hundred and eighty five isolates were available for microbiological analysis (91.5%). PCR targeting *sau1-hsdS1*, followed by MLST for positive isolates, demonstrated that 18 (17 MSSA, 1 MRSA) out of these 785 isolates (2.3%) belonged to ST398. Prevalence was the highest among MSSA (17/615, 2.8% *vs* 1/170, 0.6% for MRSA; p = .072). ST398 BSI incidence showed a marked increase during the study period (0.002 in 2007 *vs* 0.014 in 2010). Patients with ST398 BSI did not differ from patients with non ST398 BSI by gender nor age. The portal of entry was known in 14 cases (77.8%) (table 2). BSI associated with a surgical site and those related to IVD were the most frequent (4/14, 28.6% for each of the two portals of entry). A digestive portal of entry was found for three BSI cases. Digestive portals of entry were over six times more common among ST398 compared with other cases (3/14 [21.4%] for ST398 *vs* 23/653 [3.5%]; p = .014). The occurrence of death within 7 days of diagnosis was documented in all cases and two deaths were documented among the 18 cases (11.1%). Examination of patient history did not reveal any exposure to animal husbandry or retail meat products among the 18 ST398 cases.

**Table 2:** Microbiological characteristics of the 18 ST398 strains isolated from BSI cases.

Isolate code	Date and site of isolation		Characteristics of the patient		Characteristics of the BSI^2^			Antibiotic resistance^3^			*Spa-*type	PFGE patterns		
	year	Region^1^	Sex	Age	Origin (Community- or Hospital-acquired)	Portal of entry	Death within 7 days	M	E	Te		*Apa*I	*Eag*I	*Crf*9I
89	2010	C	M	49	HA	Digestive		+		+	899	A	A	A
90	2008	B	M	63	HA	Surgical Site			+		571	B	B	C
91	2010	B	M	53	CA	not known					571	B	B	C
92	2007	C	M	75	HA	IVD			+		5635	B	C	C
93	2009	C	M	58	HA	IVD			+		1451	B	C	C
94	2009	B	M	59	CA	not known	+		+		571	B	C	C
95	2009	B	M	80	HA	Skin	+		+		571	B	C	C
96	2009	FC	M	73	HA	Skin			+		6605	B	C	C
97	2010	C	M	84	HA	Surgical Site			+		1451	B	C	C
98	2010	C	M	59	HA	Digestive			+		1451	B	C	C
99	2010	FC	M	74	HA	Pulmonary			+		571	B	C	C
100	2010	C	F	69	HA	IVD			+		9378	B	C	C
101	2008	C	M	36	HA	IVD			+		571	B	D	C
102	2009	C	F	69	HA	Surgical Site			+		571	B	D	C
103	2009	C	M	68	HA	Surgical Site			+		571	B	D	C
104	2010	B	M	85	CA	not known			+		571	B	D	C
105	2008	B	M	77	CA	not known					1451	B	F	C
106	2010	C	F	<1	HA	Digestive			+		571	C	E	B

1 E Franche-Comté, C Centre and W Bretagne region

2 Intra-Venous Devices

3 Antibiotic resistance to methicillin M, erythromycin E or tetracycline Te.

**Figure 1:**
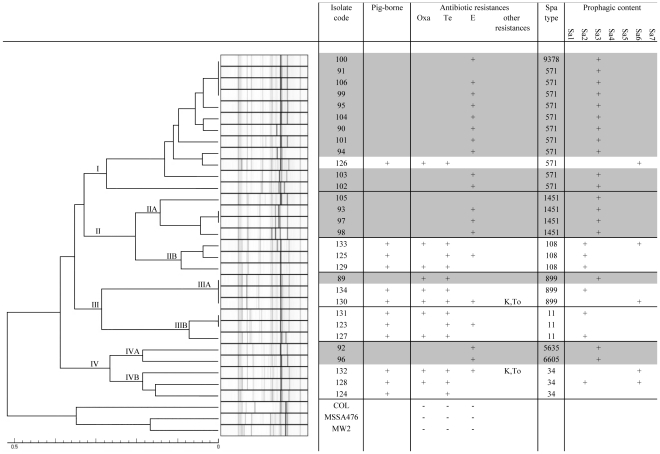
Phage content and MLVA dendrogram obtained with the 18 strains of ST398 BSI isolates and 12 strains of ST398 representing the diversity of the pig-borne clone. The dendrogram was calculated using the MLVA data obtained for18 ST398 BSI isolates, 12 pig-borne ST398 MSSA and MRSA isolates and 3 reference strains (COL, MSSA476 and MW2). On the basis of their MLVA fingerprints, the isolates segregated into different clusters. Results of antibiotic susceptibility tests [resistance to Oxa (methicillin), Te (tetracycline), E (erythromycin), K (kanamycin), To (tobramycin)], *spa*-typing, as well as determination of prophage content of Sa1int to Sa7int genes assessed by Phage integrase multiplex PCRs, are also indicated. The BSI isolates appear underlined with grey colour in the figure.

Microbiological study of the 18 ST398 isolates first revealed low diversity among antibiotic susceptibility patterns (table 2). The MRSA isolate was found resistant to tetracycline only; the 17 MSSA isolates were susceptible to all tested antibiotics (n = 2, 11.8%) or only resistant to erythromycin (n = 15, 88.2%). Significant associations were found, first, between ST398 MSSA isolates and the sole resistance to erythromycin (15/17, 88.2% for ST398 *vs* 52/598, 8.7% for nonST398 MSSA; p<.0001), and second, between ST398 MRSA and tetracycline resistance (1/1, 100% for ST398 *vs* 1/169 0.6% for non ST398 MRSA; p = .012). *Spa* typing identified seven related *spa-*types. The two *spa-*types t571 and t1451, that are infrequently associated with pig-borne isolates, were found with 9 and 4 BSI isolates, respectively. No isolate was assigned to classical pig-borne *spa-*types (i.e. t011, t034 or t108). None of the 18 ST398 isolates were positive for *tst* nor *luk* genes. Using phage integrase multiplex PCR, Sa3int phage was found in all ST398 isolates (figure 1). The prophagic content differed with the pig-borne isolates (figure 1). None of the 12 pig-borne isolates harbored Sa3int phage; one isolate didn't harbor any phage, three isolates harbored Sa6int phage, 5 isolates harbored Sa2int phage and two isolates harbored both Sa2int and Sa6int phages. MLVA distinguished clearly all the studied ST398 from the three non ST398 reference *S. aureus* isolates (figure 1). The population of ST398 isolates was divided into four divisions, named I to IV. Division I clustered the t571 MRSA pig-borne isolate and all the t571 MSSA BSI isolates. Division II was divided into two branches: IIA containing all t1451 BSI isolates, and IIB clustering all the t108 MSSA and MRSA pig-borne isolates. Division III was subdivided in IIIA and IIIB. IIIA clustered the MRSA t899 BSI isolate with the MRSA t899 pig-borne isolates, and IIIB clustered the MRSA and MSSA t011 pig-borne isolates. Division IV was subdivided into two branches. IVA comprising comprised two MSSA BSI isolates respectively characterized by *spa-*types 5635 and 6605, and IVB clustering the pig-borne MRSA t034 isolates. PFGE using *Apa*I, *Eag*I and *Crf9*I restriction enzymes revealed 3, 6 and 3 pulsotypes, respectively, mostly clustered into a homogenous division with UPGMA analysis.

## Discussion

The study confirms the emergence among French patients of BSI due to ST398, a *Staphylococcus aureus* strain formerly found primarily in association with pigs [2]. The incidence suggests a concurrent increase of MSSA BSI and ST398 BSI. In addition, BSI related to surgical site or an IVD were found preponderant among ST398 BSI cases and among increasing HA-MSSA BSI. This suggests that the increasing incidence of ST398 may play a role within the global trend toward increased incidence of MSSA BSI. More generally, however, the emergence of ST398 human infections may offer insight into the genetics of bacterial adaptation to human infection.

The precise characterization of 18 strains of ST398 revealed new characteristics of this emerging *S. aureus* clone. First and foremost, all the BSI isolates, but none of the porcine isolates, incorporated the Sa3int phage. We also showed that the 18 BSI isolates differed greatly from “classical” pig-borne isolates with respect to *spa-*type and antimicrobial susceptibility. The very prevalent pig-borne *spa-*types t011, t034 and t108 were not found in ST398 BSI isolates, belonging mainly to the *spa-*types t571 and t1451. In addition, all but one of our ST398 BSI strains had completely homogenous resistance patterns (resistant to erythromycin only). This confirms their difference from pig-borne clones, whose heterogeneity of resistance patterns and *spa-*types suggests a rapidly evolving group [15].

The ST398 BSI isolates also showed substantial diversity. Only one pair presented strictly similar *spa-*type, PFGE and MLVA patterns, and 17 were distributed into multiple distinct divisions designated by *spa-*typing and MLVA. Within all of these MLVA divisions, ST398 BSI isolates clustered at varying distances from pig-borne ST398 isolates. The single MRSA BSI isolate was very close from an MRSA t899 pig-borne isolate. The 17 MSSA ST398 BSI isolets, in contrast, in the 17 other cases, clustered at a higher genetic distance not only from pig-borne isolates, but also from other ST398 isolates within the same divisions.

This diversity may be related to the ST398's apparent shift toward human hosts. Deficiency in *Sau*I type I restriction system, resulting in hypersusceptibility to acquisition of foreign genes, have been described in ST398 clone [16]. This would account for both the rapid acquisition of genetic diversityand, in particular, to the acquisition of virulence genes for a particular host. Our observations regarding the genome content of the ST398 BSI isolates suggest that their emergence could have arisen as a consequense of recent horizontal transfers in diverse ST398 pig-borne isolates.

Horizontal transfer of phage allows bacteria to adapt to specific niches, and may lead to the emergence of new clones. Prophagic content differences have been hypothesized between pig-borne and human-adapted ST398 strains [4], [17]. In our study, the Sa3int phage distinguished all ST398 BSI isolates from all porcine isolates tested (figure 1). Pig-borne SR398 isolates were associated with other phages, ie. Sa2 and/or Sa6. The Sa3int phage harboured by all the ST398 BSI isolates includes *hlb*-converting genes that are associated with colonization and virulence in humans [12]. This lends weight to the hypothesis that a human-adapted ST398 recently evolved from pig-borne strains, and is able to accept genes conferring the ability to colonize humans, possibly by virtue of the Sa3int phage with which it is associated. This would explain the sudden ability of the emerging ST398 to infect humans in the absence of exposure to livestock or meat.

The nature of the precise gene transfer events that occurred between human and animal isolates is not elucidated. Different hypotheses for the origin of ST398 clones in different species may me formulated. The different ST398 clones could have originated from the chromosomal diversification of a common ancestor. The lateral transfer hypothesis, however, is supported by recent genomic data demonstrating that the different clones present highly similar core genome, but may be distinghished by highly distinct prophagic elements [17]. The different clones could have originated from horizontal transfer between an isolate with human origin and an isolate with animal origin. This hypothesis is supported by human to animal host switch described already within *S. aureus* species, with avian adaptation of ST5 human clone [18]. Further work should be done to better understand how the different ST398 clones emerged and spread.

This human-adapted ST398 subclone is increasingly identified in our hospitals [2], [19]. It appears to be highly receptive for horizontal gene transfer. Environmental proximity being a dominant factor in determining horizontal transfer, further acquisition of additional genetic elements harboring virulence and antibiotic resistance determinants could arise. For instance, rare cases of PVL-producing ST398 isolates have been reported, mostly from humans lacking livestock exposure [4], [20], [21]. Here, we identified one isolate of *spa-*type t899, which may result from recombination with strain belonging to the clone ST9. In *S. aureus*, variation of *spa-*type has been described previously as a result of replacement of a large part of chromosome by DNA isolates from other lineages. For instance, the epidemic ST239 has emerged after such a genetic event [22]. Presence of exogenous *spa* gene with type t899 has been reported previously in ST398 [5] suggesting that recombination would occur frequently in this genetic background. Further investigations are needed to determine if this phenomenon could drive the evolution of ST398 clones.

Much is known over pig-borne MRSA ST398 transmission between animals and humans and between humans [1]. Occupational exposure to pig results in frequent acquisition of pig-borne MRSA ST398 carriage. Until now, however, human carriage has been transient except for persons continually exposed, and human infections remained scarce, suggesting that pig-borne clones were not well-adapted to humans [23], [24]. On the other hand, recent invasive infections (*e.g.* BSI) due to ST398 are mostly associated with non-pig-borne isolates [1], [2], [4], [25]. Here, the mode of acquisition of the non pig-borne ST398 isolates by our 18 patients remains unclear. Finding *S. aureus* strains involved in human infections in meat, and reports of throat carriage among healthy subjects, suggest a potential role for food in the spread of human-adapted ST398 [25]. We found that a digestive portal of entry was much more common among ST398 compared to other *S. aureus* BSIs, lending weight to the hypothesis of foodborne transmission. Further farm-to-fork studies are required to elucidate the possible role of food, especially retail meat samples including beef, pork and chicken, as a source of human infection.
